# Complete Extruded Diet: How Does Equine Fecal Microbiota Change During Intake Adaptation?

**DOI:** 10.1111/asj.70147

**Published:** 2026-01-08

**Authors:** Bruna Caroline Franzan, Irene da Silva Coelho, Emilly Martins Ramos, Ana Rúbia Pereira de Souza, Fernando Queiroz de Almeida, Vinicius Pimentel Silva

**Affiliations:** ^1^ Animal Science Institute Universidade Federal Rural do Rio de Janeiro Rio de Janeiro Brazil; ^2^ Veterinary Institute Universidade Federal Rural do Rio de Janeiro Rio de Janeiro Brazil

**Keywords:** concentrate meal, fermentation, fiber, hindgut, horse

## Abstract

This study aimed to investigate the gradual adaptation of the fecal bacterial community and in vitro fermentative capacity of horses fed a complete extruded diet (CED). Twelve geldings weighing 370 kg were removed from a native pasture and fed coastcross hay (*Cynodon* spp) for 7 days. In the second week, horses were assigned to two groups: one group was fed exclusively with coastcross hay (HAY) for 28 days and the other group fed with weekly increases of CED (30%, 60%, and 100%) to replace HAY. Fecal samples were collected on Days 7, 14, 21, and 28 for microbiota and in vitro fermentation analyses. CED intake reduces the relative abundance of phyla Fibrobacteres, Proteobacteria, Campilobacterota, Lentisphaerae, and SR1, while increasing Verrucomicrobia and Synergistetes. The fecal bacterial diversity was maintained until Day 21 (60% CED) but declined when hay was completely withdrawn. Equine fecal microbiota is diet‐dependent; the stability of microbial diversity is more closely linked to the presence of roughage than to CED intake. Also, the inclusion of CED affects its microbial abundance and the detection of bacterial groups able to alter in vitro fermentative activity.

## 1 Introduction

The gastrointestinal microbiota of horses maintained at pasture undergoes natural changes, continuously adapting to variations in forage availability and quality driven by climatic and environmental factors (Salem et al. 2018; Fernandes et al. 2021). Nowadays, the expansion of training centers near urban areas has reduced access to grazing land (Clarke et al. 1990). This restriction also limits the area for storage of roughage foods since large areas are required for their adequate keep (NRC 2007). Consequently, dietary and feeding management practices have adapted, including reducing fiber content of the diet and increasing grain‐based processed feeds.

Low‐fiber intake combined with grain‐based diets that supply excess starch can alter the composition and stability of the intestinal microbiota and affect the pH of the equine gastrointestinal tract (Coenen and Vervuert 2010; Hansen et al. 2015). To address these challenges, high‐fiber concentrates have been formulated to meet equines' daily nutritional requirements (Miraglia et al. 2006) to maintain the equine large intestine bacterial community. Within this context, the complete extruded diet (CED) could be used as an alternative for horse feeding.

A CED is defined as the combination of all nutrients to supply the nutritional category requirements in a single product, except water (NRC 2007). Although complete diets for feeding horses have been adopted for a long time (Earle 1950), there is some evidence that their adoption can provide good results in specific situations: greater weight gain in weaned foals (Andrew et al. 2006), improved feeding management for senior horses and those with dental problems due to the reduced particle size (Argo 2016), and it has also been used in clinical events related to gastrointestinal diseases (Ralston 1990).

It is well established that several dietary factors influence the equine gut microbiota as a response to prececal digestion (Julliand and Grimm 2017; Hesta and Costa 2021). Although numerous studies in recent years have examined the relationship between diet and the equine microbiota, mainly focusing on abrupt dietary changes, the specific impact of complete diets on intestinal microbial communities remains poorly understood. Furthermore, there is a lack of standardized protocols to guide the adaptation of horses to new diets, particularly when dietary transitions involve major changes in fiber content or physical form of the feed, which may increase the risk of digestive disturbances (Hillyer et al. 2002; NRC 2007; Julliand and Grimm 2017).

This study was conducted with the aim of characterizing the fecal microbiota of horses during adaptation to intakes of a CED and their fermentative capacity in vitro simulation.

## 2 Materials and Methods

### 2.1 Ethics

All procedures were approved by the Animal Use Ethics Committee from Universidade Federal Rural do Rio de Janeiro (UFRRJ) under no. 3083.029756/2017‐10.

### 2.2 Animals

Twelve healthy Mangalarga Marchador geldings with no previous antimicrobial treatment were used in the experiment. The horses' age was 4.4 ± 1 years, and their initial body weight (BW) was 370 ± 22 kg. Clinical and hematological evaluations were performed prior to the beginning of the experimental period to confirm the animals' health status. Seven days before the start of the trial, the horses were submitted to parasite control with ivermectin and cypermethrin. On Day 0, the horses were removed from mixed pastures of *Brachiaria decumbens* ( *Urochloa decumbens* ) and bahiagrass ( *Paspalum notatum* ) and transferred to the research facilities. The horses were kept in individual stalls with free access to water and mineral salt. No antimicrobials were given to the animals during the experimental period.

### 2.3 Experimental Design

The research lasted 28 days. During the first week (Days 1–7), all horses were fed a coastcross hay diet (HAY; *Cynodon* spp. cv coastcross) ad libitum. At the beginning of the second week, the horses were randomly split into two groups. The horses in the HAY group (*n* = 6) were exclusively fed a coastcross hay diet until Day 28, whereas horses in the CED group (*n* = 6) received increasing amounts of a CED (FORAGGE HORSE, Nutratta, Brazil) to replace hay.

**FIGURE 1:**
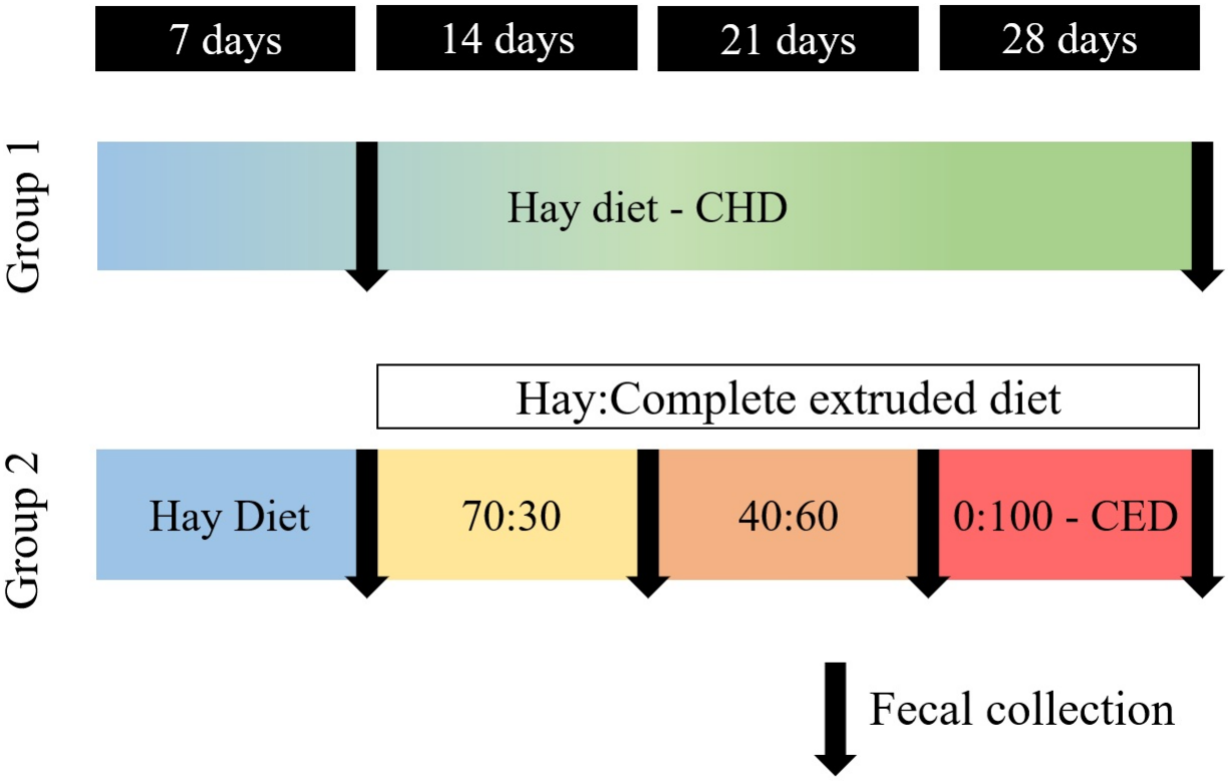
Fecal sample collection schedule for the in vitro incubation and microbiome analysis.

The horses were fed diets corresponding to 2.5% BW on a dry matter (DM) basis. The HAY:CED ratio was adjusted weekly (as fed basis) as follows: 70:30 on Day 7, 40:60 on Day 14, and 0:100% on Day 21 (Figure 1). The CED was offered in three equal meals at 06:00, 12:00, and 18:00 h, whereas hay was provided ad libitum in the hay feeder.

To address the objective of evaluating how the equine fecal microbiota changes during adaptation to CED, fecal samples were collected from horses fed either HAY or CED throughout the adaptation period as follows:

HAY group: samples collected on Days 7 and 28.

CED group: samples collected weekly on Days 7, 14, 21, and 28 corresponding to HAY:CED ratios of 100:0; 70:30; 40:60; 0:100%, respectively.

Fecal samples (300 g) were collected manually from the rectum using a single‐use glove. Immediately after collection, the samples were separately packed in sealed plastic bags and transported to the lab inside a thermal box at 39°C. The samples were at the lab within 30 min of collection. Fecal samples were immediately split into subsamples, one fraction stored at −20°C in sterilized Eppendorf (20 g) until DNA extraction, and the remaining feces were used as fecal inoculum to the in vitro fermentation assay.

The HAY and CED samples were dried in a forced draft oven for 24 h at 105°C for DM determination. The crude protein content was determined by Kjeldahl method, and ash was determined by incineration for 5 h at 600°C, as previously described (AOAC 1995). The ether extract was determined by ether solvent extraction with Soxhlet equipment, and gross energy was determined by total combustion of the samples in a bomb calorimeter (Silva and Queiroz 2006). Lignin was analyzed by oxidation with permanganate (Van Soest and Robertson 1979) as well as the content of neutral detergent fiber (NDF) and acid detergent fiber (ADF), as previously described (Van Soest et al. 1991).

### 2.4 In Vitro Fermentation Assay

An in vitro fermentation assay was performed to assess changes in the functional capacity of the microbiota during dietary adaptation. The semiautomatic gas production technique was applied, using HAY and CED as substrates. The samples were ground to 1 mm. The fermentation bottles containing the nutrient solution (Theodorou et al. 1994) and 1 g of the substrates were prepared 12 h before fecal inoculation (Franzan et al. 2018). Aliquots of 100 g of fresh feces per animal were weighed and diluted in a nutrient solution in the proportion 1:1 (weight: weight). The mixture of feces and nutrient solution was kept for 1 h on a water bath (39°C) with constant agitation and CO_2_ spraying. After 1 h, the fecal inoculum was filtered through a 45‐μm nylon cloth and used to inoculate the fermentation bottles. Three bottles were incubated for each combination of inoculum donor and substrate and kept in a water bath at 39°C for 48 h.

Gas production was measured utilizing a pressure transducer (LOGGER AG100, Universal Datalogger) at 1, 2, 3, 4, 5, 6, 7, 8, 9, 10, 11, 12, 14, 16, 18, 20, 22, 24, 27, 30, 33, 36, 39, 42, 45, and 48 h, making a total of 26 readings. The gas volume (mL g DM^−1^) was obtained using the specific equation for experimental conditions in EQUILAB‐UFRRJ (2°45′S, 43°41′W Gr and 33 m altitude): $ŷ=-0.07+3.79x+0.77x^{2}$, in which each psi (6,894.757 Pa) corresponds to 3.80 mL (Martins 2012). After 48 h of incubation, the pH was measured, and the residues of each bottle were analyzed for DM and organic matter (OM) degradation (Franzan et al. 2018).

### 2.5 DNA Extraction, Library Preparation, and Sequencing of 16s rRNA Gene

Total DNA was extracted from 0.2 g of frozen feces using the QIAamp Fast DNA Stool Mini kit (QIAGEN Ltd., United Kingdom) following the manufacturer's instructions. The V4 variable region of the bacterial 16S rRNA gene was amplified with primers 515F (GTGYCAGCMGCCGCGGTAA) and 806R (GGACTACNVGGGTWTCTAAT) (Apprill et al. 2015; Parada et al. 2016) and amplicons sequenced on the MiSeq platform paired‐end 2x250 system (Illumina, United States). The library preparation and amplicon sequencing were performed by Argonne National Lab (www.anl.gov).

### 2.6 Bioinformatics Analyses

Sequencing data demultiplexed were processed using the software Mothur v.1.41.3 (Schloss et al. 2009). Forward and reverse paired sequences were merged into contigs. Merged sequences of less than 240 or greater than 260 base pairs and sequences containing any ambiguities or more than eight homopolymers were removed from the dataset. Next, the sequences were aligned using a modified Silva database (Quast et al. 2012) and the resulting alignment was submitted to screen.seqs and filter.seqs to remove poorly aligned sequences and uninformative columns in the alignment. Then, the sequences were pre‐clustered using the command pre.cluster with parameter difs = 2. Chimeras were detected and eliminated using the commands chimera.vsearch and remove.seqs, respectively. The classify.seqs command was used with the Ribosomal Database Project version 18 (Cole et al. 2009) and a bootstrap threshold of 80. Sequences identified as derived from chloroplasts, mitochondria, Eukarya, Archaea, and not assigned to any kingdom were removed. The number of reads per sample was normalized to the sample with the lowest number of sequences. Further, the sequences were clustered into operational taxonomic units (OTU) by using the cluster.split command, with a 3% dissimilarity. Rare OTU were removed using the command split.abund. Finally, the OTU distribution, Good's coverage, the species' diversity indices Chao1 (Chao 1984), Shannon (Shannon 1948), and Simpson (Simpson 1949) as well as the rarefaction curves were calculated. The bacterial composition was evaluated using a taxonomic summary of each sample. The OTU distribution was used as an input for a nonmetric multidimensional scaling (NMDS) ordering with the Bray–Curtis similarity index.

### 2.7 Statistical Analyses

The HAY group was designed as an internal control, enabling both within‐diet and between‐diet comparisons to clarify the dynamics of fecal microbiota adaptation to CED intake. Accordingly, three sets of comparisons were performed to ensure that the observed microbial shifts could be attributed to the adaptation to CED consumption rather than to the experimental management. These included (i) horses fed HAY on Days 7 and 28; (ii) horses fed CED on Days 7, 14, 21, and 28; and (iii) comparisons between diets.

Results from in vitro fermentation assays (final gas volume, pH, and nutrient degradation, as well as outcomes from 16S rRNA gene sequencing (relative frequencies of bacterial phyla and genera, and the diversity indices) were first tested for homoscedasticity of variances and normality. Fermentation variables meeting parametric assumptions were analyzed by paired *t*‐test (*α* = 0.05) for within‐HAY temporal comparisons (Day 7 vs. Day 28), repeated‐measures ANOVA (*α* = 0.05) for comparisons across CED adaptation times, and one‐way ANOVA (*α* = 0.05) for between‐diet comparisons. When significant, means were compared using Tukey's test (*α* = 0.05).

Sequencing‐derived variables that did not meet parametric assumptions were analyzed using nonparametric tests: the Wilcoxon signed‐rank test (*p* ≤ 0.05) for within‐HAY diet temporal comparisons, the Friedman test *(p* ≤ 0.05) for repeated measures across CED adaptation times, the Mann–Whitney test (*p* ≤ 0.05) for pairwise contrasts (with Bonferroni adjustment for multiple comparisons), and the Kruskal–Wallis test (*p* ≤ 0.05) for between diets comparison.

Beta diversity (differences in structural composition between diets and adaptation times) was evaluated by PERMANOVA (*p* ≤ 0.05) based on OTU distribution. When the global PERMANOVA was significant, pairwise comparisons were performed and *p*‐values were adjusted using the Bonferroni correction (*p* ≤ 0.05).

All procedures were performed using the Past4.07 statistical package (Hammer and Harper 2001).

### 2.8 Accession Number of the Nucleotide Sequences

The data generated were deposited in the NCBI *Sequence Read Archive* (SRA) and are available under the bioproject accession number PRJNA640129.

## 3 Results

### 3.1 Chemical Composition of Feed and Nutrient Intakes

The results of the chemical analysis of the feeds are presented in Table 1. The average DM intake was 25 g DM kg^−1^ BW for the diets tested (Table 2), and this amount meets the nutritional requirements of maintenance horses (NRC 2007).

**TABLE 1:** Chemical composition of coastcross hay and complete extruded diet, as dry matter (DM) basis.

	Feed	
Item	Coastcross hay	Complete extruded diet
Dry matter, g/kg	900	888
Chemical composition, g/kg DM		
Ash	58	46
Ether extract	23	33
Crude protein	157	114
Neutral detergent fiber	823	446
Acid detergent fiber	392	255
Cellulose	319	200
Lignin^a^	73	55
Nonfibrous carbohydrates^b^	0	362
Energy content		
Gross energy, kcal/kg DM	4421	4572
Digestible energy, kcal/kg DM^c^	1740	2710

^a^ Lignin determined by permanganate oxidation.

^b^ Nonfibrous carbohydrates = 100 − (% crude protein + % neutral detergent fiber + % ether extract + % ash).

^c^ Estimated Digestible Energy (NRC 2007) = 2118 + 12.18 × (% crude protein) − 9.37 × (% acid detergent fiber) − 3.83 × (% hemicellulose) + 47.18 × (% ether extract) + 20.35 × (% nonfibrous carbohydrates) − 26.3 × (% ash).

**TABLE 2:** Estimated nutrient intake of horses fed a coastcross hay diet (HAY) in two adaptation times or an increased inclusion of complete extruded diet to replace the HAY.

Item, g/day	HAY	Complete extruded diet inclusions (%)			
		0	30	60	100
Ash	580	580	544	508	460
Ether extract	230	230	260	290	330
Crude protein	1570	1570	1441	1312	1140
Neutral detergent fiber	8230	8230	7099	5968	4460
Acid detergent fiber	3920	3920	3509	3098	2550
Cellulose	3190	3190	2833	2476	2000
Nonfibrous carbohydrates^a^	0	0	1086	2172	3620

^a^ Nonfibrous carbohydrates = 100 − (% crude protein + % neutral detergent fiber + % ether extract + % ash).

### 3.2 Sequencing Metrics

Thirty fecal samples yielded 793,968 sequences. After alignment and quality filtering, 599,512 high‐quality non‐chimeric sequences remained, of which 597,232 were classified as Bacteria domain (75.22% of the original number of sequences). After data normalization, 439,200 sequences (14,640 per sample) were clustered into 4120 OTU (3% dissimilarity threshold) and used for downstream calculations. The number of subsampled sequences was considered adequate to represent the bacterial diversity present in the fecal samples, since Good's coverage estimates on the normalized OTU table indicated mean coverage being 98.75%. Approximately 90% of the sequences were identified to the phylum level. Additionally, 59% of the sequences were classified at the class level, 55% at the order level, 41% at the family level, and only 17% at the genus level. In all, 223 groups in the lowest taxonomic division were observed, wherein 18 bacterial phyla were detected, which encompassed at least 33 different taxonomic classes, 51 orders, 79 families, and 143 genera.

### 3.3 Alpha‐Diversity

Results regarding fecal species diversity for all comparisons are presented in Table 3. During the first week, when all horses were fed exclusively HAY, similar diversity indices were observed among the animals. Likewise, within the HAY group, no significant differences (*p* > 0.05) were observed in fecal diversity indices between Days 7 and 28.

**TABLE 3:** Alpha diversity indices of fecal bacterial community in horses fed coastcross hay (HAY) in two adaptation times or an increased inclusion of complete extruded diet (CED) to replace the HAY.

	HAY			Complete extruded diet inclusions (%)^a^					Between diets^b^		
Index	7	28	*p*	0	30	60	100	*p*	HAY	CED	*p*
Number of OTU	1223	1218	0.75	1319*	1300*	1327*	777*	< 0.01	1218*	777*	< 0.01
Chao1	1770.8	1782.2	0.75	1885.9*	1919.6*	1893.5*	1184.4*	< 0.01	1782.2*	1184.4*	< 0.01
Shannon	5.45	5.48	0.75	5.61*	5.77*	5.68*	4.58*	< 0.01	5.48*	4.58*	< 0.01
Simpson	0.014	0.014	0.602	0.012*	0.008*	0.009*	0.032*	< 0.01	0.014*	0.032*	< 0.01

^a^ Means with different letters within “Complete extruded diet inclusions” differ significantly (*p* < 0.05) by the Mann–Whitney test.

^b^ Means with different letters within “Between diets” significantly (*p* < 0.05) by the Kruskal–Wallis test.

* Indicate statistical differences among means.

In contrast, during the stepwise adaptation to CED, no significant differences in alpha‐diversity were observed when horses received 0% (Day 7), 30% (Day 14), and 60% (Day 21) CED replacing HAY. However, after complete replacement (100% of CED on day 28), fecal samples showed lower Chao and Shannon indices and a higher Simpson index compared with the previous weeks (*p* < 0.01).

At the end of the adaptation period (Day 28), comparisons between diets revealed that horses fed HAY had significantly higher alpha‐diversity than those fed with CED (*p* < 0.01).

### 3.4 Taxonomic Composition of Bacterial Communities

**FIGURE 2:**
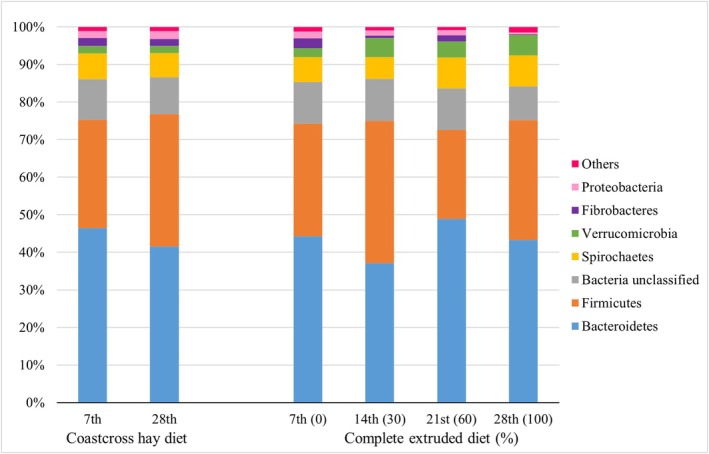
Fecal bacterial phyla with relative abundance greater than 1% in horses fed a Coastcross hay diet (HAY) on Days 7 and 28 or in horses fed increasing inclusions of a complete extruded diet, 0%, 30%, 60%, and 100% on Days 7, 14, 21, and 28, respectively.

**FIGURE 3:**
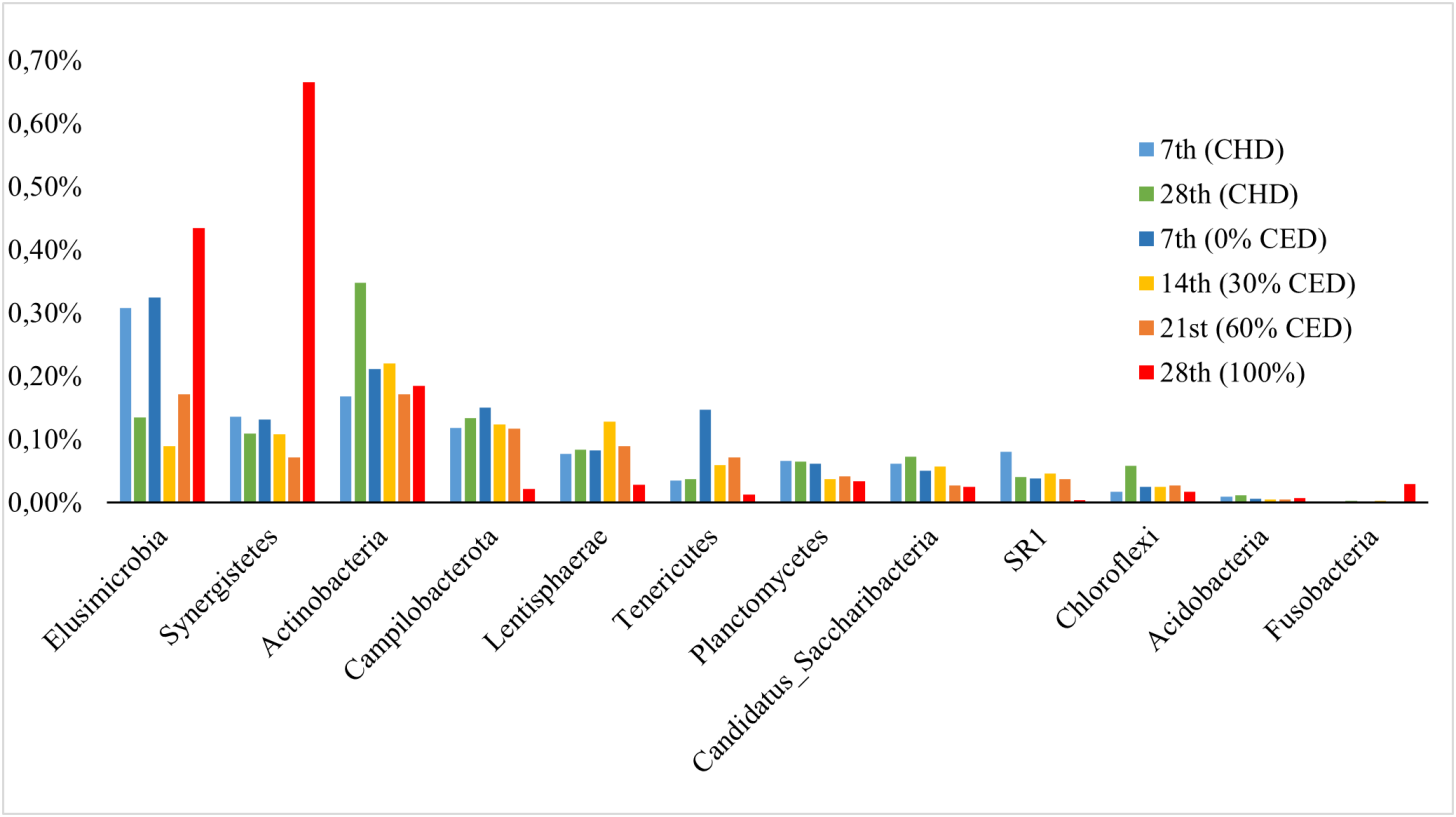
Fecal bacterial phyla with relative abundance less than 1% in horses fed a coastcross hay (HAY) on Days 7 and 28 or in horses fed increasing inclusions of complete extruded diet (CED), 0%, 30%, 60%, and 100%, on Days 7, 14, 21, and 28, respectively.

The phyla Bacteroidetes and Firmicutes represented approximately 75% of the bacterial community in all fecal samples (Figure 2). The following phyla each represented less than 1% of relative abundance: Elusimicrobia, Synergistetes, Actinobacteria, Campilobacterota, Lentisphaerae, Tenericutes, Planctomycetes, Candidatus Saccharibacteria, SR1, Chloroflexi, Acidobacteria, and Fusobacteria. Together, these phyla represented approximately 2% of the bacterial community (Figure 3). Among the 18 bacterial phyla identified, Fusobacteria was not detected in the fecal samples collected on Day 7.

The composition of the fecal bacterial community remained unchanged in horses fed HAY between Days 7 and 28 (Figure 2). However, the relative abundance of the phylum Elusimicrobia was significantly higher on Day 7 than on Day 28 (*p* = 0.03), whereas Actinobacteria was significantly higher on Day 28 than on Day 7 (*p* = 0.03). Significant differences at the genus level between fecal samples collected on Days 7 and 28 are presented in Table S1.

Relative abundance analysis revealed significant differences across CED adaptation for the phyla Firmicutes (*p* = 0.05), Verrucomicrobia (*p* = 0.04), Fibrobacteres (*p <* 0.01), Proteobacteria (*p* = 0.02), Synergistetes (*p* < 0.01), Campilobacterota (*p* = 0.02), Lentisphaerae (*p* = 0.04), and SR1 (*p =* 0.02) across CED adaptation. Firmicutes increased with the inclusion of CED on Day 14 but decreased to its lowest level when horses were fed with 60% CED on Day 21, with no significant difference between Days 7 and 28. Fibrobacteres decreased with the inclusion of CED on Day 14, increased on Day 21, and reached the lowest level with 100% CED on Day 28. Verrucomicrobia increased with the inclusion of CED, but no significant differences were observed among Days 14, 21, and 28. Horses fed with 100% CED on Day 28 showed a significant increase in Synergistetes (Figure 3), whereas Proteobacteria, Campilobacterota, Lentisphaerae, and SR1 showed significant decreases on the same day. At the genus level, different patterns of change in relative abundance were observed during CED adaptation. Significant differences are shown in Table S2.

On Day 28, no significant differences were observed in the relative abundance of the most abundant phyla Bacteroidetes and Firmicutes between diets (*p* > 0.05; Figure 3). However, the abundance of Fibrobacteres (*p <* 0.01), Proteobacteria (*p <* 0.01), Campilobacterota (*p =* 0.02), Lentisphaerae (*p =* 0.02), Planctomycetes (*p =* 0.04), SR1 (*p =* 0.03), and Chloroflexi (*p =* 0.02) was higher in horses fed HAY than in those fed CED. In contrast, Verrucomicrobia (*p <* 0.01) and Synergistetes (*p <* 0.01) were more abundant in horses fed CED. Significant differences observed at the genus level between diets on Day 28 are shown in Table S3.

### 3.5 Multivariate Analysis of OTU Distribution

**FIGURE 4:**
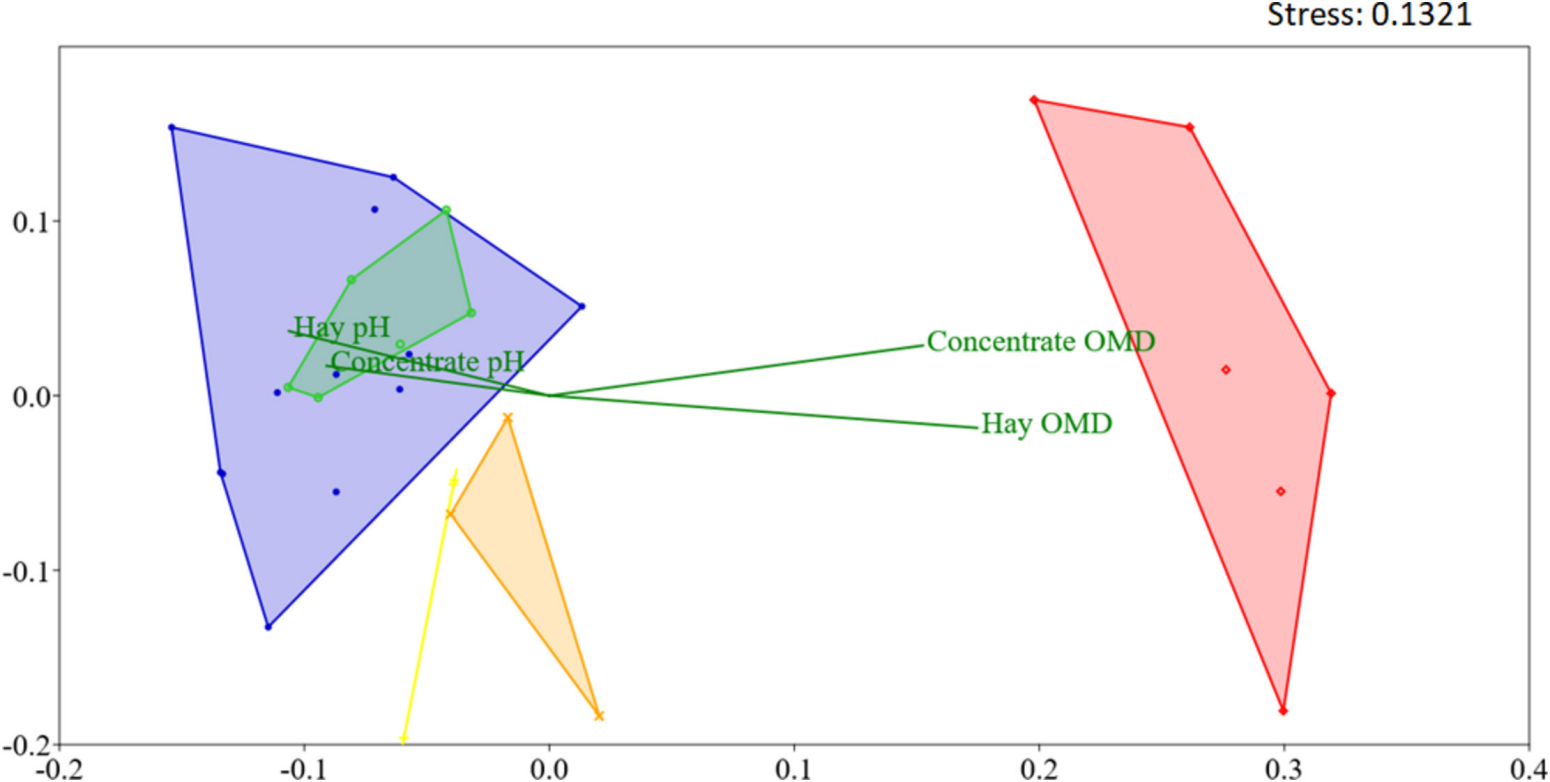
Nonmetric multidimensional scaling base Bray–Curtis similarity index based on OTU distribution. Blue: fecal samples from all horses fed a coastcross hay diet (HAY) on Day 7; green: horses fed HAY on Day 28; yellow: horses fed 30% complete extruded diet (CED) on Day 14; orange: horses fed 60% CED on Day 21; red: horses fed 100% CED on Day 28. Fermentation parameters pH and organic matter degradation (OMD) of hay and concentrate substrates are plotted as vectors. Stress values scale ranging from 0 to 1.

Analysis of OTU distribution indicated that the diet had a significant effect on the fecal bacterial community structure (PERMANOVA; *p* < 0.001). NMDS revealed the relationships between bacterial composition, diets, and time of adaptation (Figure 4).

Fecal samples collected from all horses on Day 7 showed bacterial communities similar to those of horses fed some level of CED on Days 14 and 21. In addition, fecal samples collected on Day 28 from HAY group horses revealed a bacterial community distinct from that observed in horses fed CED on Days 14, 21, and 28. Therefore, on Day 28, the two diets resulted in significantly different bacterial communities.

### 3.6 In Vitro Fermentation Assay

As all animals were fed HAY until Day 7, no differences were observed in the fermentation variables evaluated: final gas production, DM degradation, OM degradation, and pH.

The fecal inoculum collected from horses fed HAY showed greater fermentation capacity on Day 28 when HAY was used as the substrate (*p* < 0.05), as evidenced by increases in the final gas volume, DM and OM degradation, and pH (Table 4). Also, significant differences were observed in total gas production and final residual pH obtained from samples collected on Days 7 and 28 when the concentrate was used as substrate (*p* < 0.05). However, no significant differences were observed in nutrient degradation (*p >* 0.05).

**TABLE 4:** Mean values of in vitro fermentation variables from substrate coastcross hay and complete extruded diet incubated with feces from horses fed a coastcross hay (HAY) in two adaptation times or increased inclusions of complete extruded diet (CED) to replace the HAY.

	HAY^a^			Complete extruded diet inclusions (%)^b^					Between diets^c^		
Substrate	7	28	*p*	0	30	60	100	*p*	CHD	CED	*p*
Coastcross hay											
Total gas production, mL/g DM	51.1*	80.6*	< 0.001	55.3*	72.7*	68.3*	87.2*	< 0.001	80.6	87.2	0.22
Dry matter degradation, mg/g	226*	357*	< 0.001	239*	362*	319*	393*	< 0.001	357	393	0.06
Organic matter degradation, mg/g	216*	370*	< 0.001	235*	369*	303*	421*	< 0.001	370*	421*	0.01
Final residual pH	6.53*	6.38*	0.04	6.56*	6.36*	6.51*	6.36*	< 0.001	6.38	6.36	0.40
Complete extruded diet											
Total gas production, mL/g DM	135.2*	159.2*	0.04	127.7*	140.4*	120.1*	159.8*	< 0.001	159.2	159.8	0.94
Dry matter degradation, mg/g	426	443	0.55	406*	512*	422*	504*	< 0.001	443*	504*	0.01
Organic matter degradation, mg/g	433	475	0.22	419*	531*	424*	551*	< 0.001	475*	551*	0.01
Final residual pH	6.40*	6.25*	0.02	6.40*	6.17*	6.33*	6.24*	< 0.001	6.25	6.24	0.59

^a^ Means with different letters within “HAY” differ significantly (*p* < 0.05) by paired *t*‐test.

^b^ Means with different letters within “Complete extruded diet inclusions” differ significantly (*p* < 0.05) by the Tukey test.

^c^ Means with different letters within “Between diets” differ significantly (*p* < 0.05) by the Fisher test.

* Indicate statistical differences among means.

When CED was included in the diet, gas production as well as DM and OM degradation increased significantly when HAY was used as the substrate (*p* < 0.001). However, the final pH of the liquid residue from the bottles containing HAY was lower on Days 14 and 28 than on the other days (*p* < 0.001), when horses were fed with 30% and 100% CED, respectively. For fermentation with CED as substrate, a significant difference was observed in the final gas volume and nutrient degradation (*p* < 0.001), with higher means observed on Days 14 and 28. However, the final pH values significantly decreased on Day 14 (*p* < 0.001).

After 28 days of exclusive intake of the experimental diets, fecal inoculum from horses fed with CED showed higher OM degradation during HAY fermentation (*p* = 0.01). When CED was fermented, fecal inoculum from the CED group also showed higher DM and OM degradation (*p* = 0.01).

## 4 Discussion

We decided to use horses of the same breed, age, and breeding conditions (management, facilities, pasture) to ensure the observed effects on microbiota could really be associated with dietary treatments. At the beginning of the study, all horses were submitted to the same management and feeding, first at the native pasture and then fed with HAY until Day 7 to establish a common baseline. We hypothesized that all horses (*n* = 12) would have a similar fecal bacterial community structure at this point. In addition, the HAY group served as a temporal internal control to exclude the possibility that management during the experimental period was responsible for changes in the microbiota.

After 28 days of HAY intake, further changes in the fecal microbiota composition were observed, with an increase in the phylum Actinobacteria and Firmicutes‐related taxa at genus level (e.g., *Firmicutes_unclassified*, *Planococcaceae_unclassified*, *Saccharofermentans*, *Bacillales_unclassified*, and *Eubacteriaceae_unclassified*). These shifts suggest an enhanced fermentative capacity of the community, as members of Firmicutes are recognized as major fiber degraders and producers of short‐chain fatty acids in the equine gut (Dougal et al. 2012; Morrison and Preston 2016), and Actinobacteria has been associated with bacterial communities that induce high acetate production, consequently leading to the generation of blood metabolites related to lipid metabolism (Plancade et al. 2019; Langner et al. 2020). In fact, the fermentative capacity of the fecal inoculum improved after 28 days. Conversely, some genera with potential detrimental activity decreased, such as *Desulfovibrionaceae_unclassified* and *Oscillibacter*. Moreover, the reduction of *Bacteroidaceae_unclassified* and *Cellulosilyticum* indicates that Bacteroidetes and some cellulolytic representatives may have been outcompeted by Firmicutes groups better adapted to the offered substrate. Overall, these shifts suggest a microbial transition towards a more specialized community (Salem et al. 2018; Fernandes et al. 2021).

Maintaining gut homeostasis is essential for equine nutrition and health and is closely linked to substrate availability to the microbiota (Daly et al. 2012). Cereal grains are commonly used when energy requirements increase, but abrupt transitions can disrupt the intestinal ecosystem, increasing lactate‐producing bacteria (e.g., *Streptococcus* and *Lactobacillus*) and reducing fibrolytic populations due to pH decline in the cecum and colon (De Fombelle et al. 2001; van den Berg et al. 2013). Previous research has demonstrated that sudden change from hay to a CED rapidly reduces bacterial diversity, alters microbiota structure, and lowers fecal pH (Franzan et al. 2022). To avoid such effects, we adopted a gradual replacement of hay with CED within 21 days. This protocol was designed based on reports exploring the relationship between dietary changes, their impact on the intestinal microbiota, and the increased risk of large intestine disorders (De Fombelle et al. 2001; Curtis et al. 2019), as well as practical feeding considerations. Textbooks recommend gradual transitions, mostly supported by high‐fiber versus high‐starch diets studies and epidemiological evidence of colic risk. The NRC (2007) also emphasized gradual dietary changes, citing Lewis (1995), who suggested that concentrate increments of only 0.2 to 0.3 kg/day to provide sufficient time for adaptation; however, the NRC also noted that this recommendation was not based on experimental evidence. In addition, the risk of colic is highest within the first 7 days following the inclusion of hydrolysable carbohydrates in the equine diet, declining after 14 days (Hillyer et al. 2002). Furthermore, the inclusion of hydrolysable carbohydrates exceeding 0.4% BW overwhelms the prececal starch digestion capacity (Potter et al. 1992; Lopes et al. 2004), and digestive adaptations such as increased glucose transporters may take up to two months (Dyer et al. 2009). Nevertheless, it seemed to us that diet adaptation over 2 months is not practical in horse feeding. Thus, we determined that 1 month would be adequately applied in practice.

We estimate that progressive inclusion of CED over the experimental weeks increased NFC intake to 0.91, 1.81, and 3.02 g/kg BW/meal at 30%, 60%, and 100% substitution, respectively. These dietary changes shaped distinct microbial ecosystems with several phyla (Proteobacteria, Campilobacterota, Lentisphaerae, Tenericutes, and SR1) persisting only when hay was present, even as few as 10 g DM/kg BW/day (offered until Day 21). In contrast, Verrucomicrobia abundance increased with CED intake, regardless of the quantity consumed (30%, 60%, or 100%), while Synergistetes peaked on Day 28 after hay withdrawal. In addition, hay seems to be required for maintenance of microbial diversity, because the alpha diversity indices (Chao1, Shannon) remained stable during partial hay inclusion (Days 7–21), but decreased sharply once hay was removed (Day 28), increasing Simpson's dominance. This indicates that the loss of roughage, rather than NFC increased per se, was the main driver of reduced community stability. Therefore, it is necessary to separate the effects of high starch inclusion, herein estimated by the NFC content in the diet, and the absence of roughage on the stability of the equine fecal microbiome. Future studies should thoroughly evaluate the microbiome of horses fed 3.0 g NFC/kg BW/meal associated with a roughage intake.

When HAY was used as the substrate, the fecal inoculum from horses fed CED showed a progressive increase in fermentative capacity, accompanied by a reduced pH of the residual liquid on Days 14 and 28, with 30% and 100% CED, respectively. In contrast, gas production did not increase when CED was used as the substrate, but the same effect on pH was observed on Days 14 and 28. As reported by Zeyner et al. (2004), reduced roughage intake with concentrate feeding lowers fecal buffering capacity. Additionally, dietary starch intake promotes adaptive changes in the equine small intestine by upregulating glucose transport mechanisms. The greater expression of SGLT1 in the small intestine depends on the time (up to 2 months) and quantity of starch (Dyer et al. 2009). We evaluated the relative abundance of lactic acid–producing genera (*Weissella*, *Streptococcus*, *Ligilactobacillus*, and *Limosilactobacillus*) to understand the differences observed in the fecal buffering capacity during the animals' adaptation to the CED. These genera abundance patterns closely followed the changes in pH. These findings indicate that between Days 14 and 21, the microbiota can adapt to reduced glucose input to the hindgut. Therefore, diet transitions should start with less than 30% concentrate to prevent early loss of intestinal buffering capacity.

After 28 days of diet adaptation, the fecal bacterial communities of the two groups diverged. Horses fed exclusively with HAY maintained a relatively uniform microbial community, which differed from those observed in animals receiving any level of CED. In another hand, beyond the intestinal pH decrease associated with high NFC intake, competition between microorganisms adapted to the available substrate compared with those that are obligate fibrolytic (Daly et al. 2012) contributed to a reduction in the relative abundance of six phyla. Unlike our results, a greater abundance of the phylum Verrucomicrobia has previously been reported in forage‐fed horses compared with those fed high‐starch or haylage diets (Julliand and Grimm 2017; Warzecha et al. 2017). Furthermore, an increased abundance of this phylum has been associated with obesity status in horses (Elzinga et al. 2016) and appears to be linked to beneficial intestinal functions, including immune modulation, maintenance of the mucosal barrier, and potentially protection against intestinal inflammation (Lindenberg et al. 2019). Because the HAY group was included as an internal control for experimental management, it is reasonable to attribute the increased abundance of this phylum in the CED group to dietary effects. It is possible that CED intake itself, or the change in feeding management involving three daily meals of CED, stimulated the increase of this phylum in the horses CED group.

Although the importance of fiber consumption is well known in equine nutrition, so far, daily requirements have not been fully established. Hintz (Hintz 2000) suggested that a minimum of 24% NDF or 14% ADF in the total diet is adequate for maintaining gastrointestinal health, while Drogoul et al. (2001) recommended a minimum of 50% of NDF to prevent intestinal disorders in horses fed with cereal grain diets. The exclusive CED intake did meet Hintz's recommendation, as it contained 45% NDF and 25% ADF. However, the exclusion of HAY increased the abundance of the phylum *Fusobacteria* in horses fed CED. Higher relative abundance of this phylum has been associated with colitis (Costa et al. 2012) and diarrhea (Rodriguez et al. 2015). None of the horses evaluated did present any gastrointestinal disorders throughout the experimental period; nevertheless, it cannot be confirmed that long‐term feeding with CED would preserve intestinal microbial community structure and, consequently, gastrointestinal health.

The present study only evaluated a short overview of CED intake adaptation. Therefore, to better evaluate the nutritional and safety of CED use as a feed strategy, it would be interesting to investigate the long‐term effect. It would also be interesting to evaluate different environmental challenges to confirm the changing pattern of the fecal microbiome, including the use of other horse categories, such as weaned foals, geriatric horses with dental problems, horses under postoperative feeding management, and those submitted to exercise regimens. In addition, future research should investigate the impact of this kind of diet on unfamiliar equine microbiome communities, such as fungi, archaea, and protozoa. For instance, barcoded amplicon sequencing combined with qPCR can simultaneously profile bacteria, archaea, and fungi (Edwards et al. 2020). More recently, long‐read sequencing has proven useful to capture both bacterial and anaerobic fungal diversity under different dietary conditions (Wunderlich et al. 2025). These methods would provide a more complete understanding of how extruded diets influence the equine gut ecosystem.

In conclusion, our results demonstrate that equine fecal bacterial communities are strongly shaped by dietary composition and the adaptation period to diets. The stability of fecal microbial diversity appears to be more closely associated with the presence of roughage rather than the increase in CED. These findings highlight the importance of gradual dietary transitions, as microbial fermentative activity and diversity depend on adaptation to the substrates reaching the hindgut. Taken together, these findings suggest that bacterial diversity could serve as a key indicator for developing adaptation protocols or, at the very least, for estimating the minimum time required for dietary transitions.

## Conflicts of Interest

The authors declare no conflicts of interest.

## Supporting information


**Table S1:** Relative abundance (%) of genera with a significant difference in the comparison between fecal samples from horses fed with coastcross hay on Days 7 and 28.

**Table S2:** Relative abundance (%) of genera with a significant difference in fecal samples from horses fed increasing inclusions of complete extruded diet (CED) replacing the coastcross hay.

**Table S3:** Relative abundance (%) of genera with a significant difference in the comparison between feces of horses fed coastcross hay or complete extruded diet on Day 28.
