# Supplementary material for: Complete Extruded Diet: How Does Equine Fecal Microbiota Change During Intake Adaptation?
Source: Anim Sci J. 2026 Jan 8;97(1):e70147. doi: 10.1111/asj.70147 (PMC12782053; doi:10.1111/asj.70147)
Supplement: Supplementary file 1 — Table S1: Relative abundance (%) of genera with a significant difference in the comparison between fecal samples from horses fed with coastcross hay on Days 7 and 28. [file ASJ-97-e70147-s003.docx]

**Table S1.** Relative abundance (%) of genera with a significant difference in the comparison between fecal samples from horses fed with Coastcross hay on day 7 and day 28

| **Genus** | **Day 7** | **Day 28** | ***P value*** |
| --- | --- | --- | --- |
| Firmicutes_unclassified | 1.84 | 2.61 | 0.0278 |
| Planococcaceae_unclassified | 0.32 | 2.54 | 0.0464 |
| *Saccharofermentans* | 0.47 | 0.93 | 0.0277 |
| Bacillales_unclassified | 0.07 | 1.27 | 0.0431 |
| Desulfovibrionaceae_unclassified | 0.34 | 0.10 | 0.0464 |
| Alphaproteobacteria_unclassified | 0.19 | 0.06 | 0.0464 |
| *Oscillibacter* | 0.16 | 0.08 | 0.0277 |
| Bacteroidaceae_unclassified | 0.15 | 0.07 | 0.0464 |
| Coriobacteriia_unclassified | 0.03 | 0.09 | 0.0464 |
| *Elusimicrobium* | 0.09 | 0.03 | 0.0458 |
| Planctomycetacia_unclassified | 0.05 | 0.03 | 0.0277 |
| Eubacteriaceae_unclassified | 0.02 | 0.05 | 0.0464 |
| *Solibacillus* | 0.00 | 0.05 | 0.0422 |
| *Cellulosilyticum* | 0.024 | 0.008 | 0.0260 |
| *Paraeggerthella* | 0.001 | 0.009 | 0.0394 |
| Solirubrobacterales_unclassified | 0.005 | 0.000 | 0.0455 |
