# Supplementary material for: Complete Extruded Diet: How Does Equine Fecal Microbiota Change During Intake Adaptation?
Source: Anim Sci J. 2026 Jan 8;97(1):e70147. doi: 10.1111/asj.70147 (PMC12782053; doi:10.1111/asj.70147)
Supplement: Supplementary file 2 — Table S2: Relative abundance (%) of genera with a significant difference in fecal samples from horses fed increasing inclusions of complete extruded diet (CED) replacing the coastcross hay. [file ASJ-97-e70147-s001.docx]

**Table S2.** Relative abundance (%) of genera with a significant difference in fecal samples from horses fed increasing inclusions of complete extruded diet (CED) replacing the Coastcross hay

|  | **Increased inclusions of CED** | | | |  |
| --- | --- | --- | --- | --- | --- |
| **Genus** | **0%** | **30%** | **60%** | **100%** | ***P value*** |
| Clostridiales_unclassified | 3.63^a^ | 3.44^a^ | 2.88^a^ | 1.60^b^ | 0.0064 |
| Proteobacteria_unclassified | 0.40^a^ | 0.34^a^ | 0.35^a^ | 0.10^b^ | 0.0085 |
| *Vampirovibrio* | 0.25^a^ | 0.18^a^ | 0.21^a^ | 0.01^b^ | 0.0077 |
| SR1_unclassified | 0.038^a^ | 0.046^a^ | 0.037^a^ | 0.003^b^ | 0.0228 |
| *Pseudobutyrivibrio* | 0.15^a^ | 0.28^a^ | 0.26^a^ | 0.05^b^ | 0.0133 |
| *Campylobacter* | 0.12^a^ | 0.12^a^ | 0.09^a^ | 0.02^b^ | 0.0234 |
| *Lacrimispora* | 0.041^a^ | 0.034^a^ | 0.016^a^ | 0.000^b^ | 0.0316 |
| *Anaerotignum* | 0.042^a^ | 0.018^a^ | 0.007^a^ | 0.000^b^ | 0.0206 |
| *Blautia* | 0.020^a^ | 0.016^a^ | 0.011^a^ | 0.000^b^ | 0.0468 |
| *Agathobacter* | 0.008^a^ | 0.007^a^ | 0.014^a^ | 0.000^b^ | 0.0165 |
| Pasteurellaceae_unclassified | 0.028^a^ | 0.034^a^ | 0.016^a^ | 0.001^b^ | 0.0423 |
| Planococcaceae_unclassified | 0.31^a^ | 0.77^a^ | 0.03^b^ | 0.01^b^ | 0.0064 |
| *Sporobacter* | 0.037^a^ | 0.046^a^ | 0.011^b^ | 0.011^b^ | 0.0158 |
| Kiloniellaceae_unclassified | 0.023^a^ | 0.009^a^ | 0.000^b^ | 0.002^b^ | 0.0153 |
| ***Ligilactobacillus*** | **0.50^a^** | **0.92^a^** | **0.11^b^** | **0.18^b^** | **0.0421** |
| *Fibrobacter* | 2.70^a^ | 0.71^b^ | 1.60^a^ | 0.14^c^ | 0.0061 |
| *Clostridium*_XlVa | 0.053^a^ | 0.016^b^ | 0.041^a^ | 0.000^c^ | 0.0087 |
| *Anaeroplasma* | 0.138^a^ | 0.021^b^ | 0.044ª | 0.001^c^ | 0.0338 |
| *Cellulosilyticum* | 0.032^a^ | 0.011^b^ | 0.046^a^ | 0.003^c^ | 0.0277 |
| Alphaproteobacteria_unclassified | 0.15^a^ | 0.07^b^ | 0.11^a^ | 0.06^b^ | 0.0395 |
| *Anaerosporobacter* | 0.14^a^ | 0.02^b^ | 0.10^a^ | 0.00^b^ | 0.0042 |
| *Helicobacter* | 0.029^a^ | 0.000^b^ | 0.023^a^ | 0.003^b^ | 0.0377 |
| Deltaproteobacteria_unclassified | 0.008^a^ | 0.000^b^ | 0.007^a^ | 0.000^b^ | 0.0342 |
| *Intestinimonas* | 0.041ª | 0.000^b^ | 0.009^b^ | 0.008^b^ | 0.0126 |
| Desulfovibrionaceae_unclassified | 0.28^a^ | 0.13^b^ | 0.06^b^ | 0.03^b^ | 0.0028 |
| Bacillaceae_1_unclassified | 0.008^a^ | 0.000^b^ | 0.002^b^ | 0.006^b^ | 0.0500 |
| ***Weissella*** | **0.03^b^** | **1.74^a^** | **1.02^a^** | **1.10^a^** | **0.0116** |
| Subdivision5_unclassified | 2.25^b^ | 4.91^a^ | 4.18^a^ | 5.36^a^ | 0.0258 |
| ***Streptococcus*** | **0.09^b^** | **0.90^a^** | **0.57^a^** | **0.41^a^** | **0.0098** |
| Synergistaceae_unclassified | 0.13^b^ | 0.11^b^ | 0.06^b^ | 0.66^a^ | 0.0061 |
| *Saccharofermentans* | 0.56^b^ | 1.16^b^ | 0.41^b^ | 3.86^a^ | 0.0278 |
| Selenomonadaceae_unclassified | 0.31^b^ | 0.44^b^ | 0.28^b^ | 1.89^a^ | 0.0085 |
| *Mogibacterium* | 0.14^b^ | 0.10^b^ | 0.07^b^ | 0.24^a^ | 0.0023 |
| Bacillales_unclassified | 0.099^b^ | 0.165^a^ | 0.002^c^ | 0.000^c^ | 0.0024 |
| *Solibacillus* | 0.003^b^ | 0.011^a^ | 0.002^b^ | 0.000^b^ | 0.0288 |
| *Oligosphaera* | 0.064^b^ | 0.126^a^ | 0.066^b^ | 0.022^b^ | 0.0301 |
| *Pseudomonas* | 0.000^b^ | 0.007^a^ | 0.002^b^ | 0.000^b^ | 0.0432 |
| *Prevotella* | 0.27^b^ | 0.82^a^ | 2.50^a^ | 0.48^b^ | 0.0173 |
| *Fournierella* | 0.013^b^ | 0.034^a^ | 0.032^a^ | 0.005^b^ | 0.0414 |
| ***Limosilactobacillus*** | **0.00^c^** | **0.21^a^** | **0.01^c^** | **0.09^b^** | **0.0170** |
| *Succinivibrio* | 0.00^c^ | 0.02^b^ | 0.41^a^ | 0.14^b^ | 0.0028 |
| *Bacteroides* | 0.001^c^ | 0.009^b^ | 0.005^b^ | 0.176^a^ | 0.0332 |
| *Frisingicoccus* | 0.000^b^ | 0.002^b^ | 0.021^a^ | 0.010ª | 0.0296 |
| *Synergistes* | 0.000^b^ | 0.000^b^ | 0.005^a^ | 0.008ª | 0.0396 |
