# Supplementary material for: Complete Extruded Diet: How Does Equine Fecal Microbiota Change During Intake Adaptation?
Source: Anim Sci J. 2026 Jan 8;97(1):e70147. doi: 10.1111/asj.70147 (PMC12782053; doi:10.1111/asj.70147)
Supplement: Supplementary file 3 — Table S3: Relative abundance (%) of genera with a significant difference in the comparison between feces of horses fed coastcross hay or complete extruded diet on Day 28. [file ASJ-97-e70147-s002.docx]

**Table S3.** Relative abundance (%) of genera with a significant difference in the comparison between feces of horses fed Coastcross hay or complete extruded diet on day 28

| **Genus** | **Coastcross hay** | **Complete extruded diet** | ***P value*** |
| --- | --- | --- | --- |
| Subdivision5_unclassified | 1,81 | 5,36 | 0,0039 |
| Clostridiales_unclassified | 3,19 | 1,60 | 0,0039 |
| Firmicutes_unclassified | 2,61 | 1,61 | 0,0163 |
| Planococcaceae_unclassified | 2,54 | 0,01 | 0,0071 |
| Selenomonadaceae_unclassified | 0,50 | 1,89 | 0,0104 |
| *Fibrobacter* | 1,78 | 0,14 | 0,0039 |
| Bacillales_unclassified | 1,27 | 0,00 | 0,0021 |
| *Escherichia*/*Shigella* | 1,09 | 0,06 | 0,0081 |
| Spirochaetaceae_unclassified | 0,75 | 0,37 | 0,0250 |
| *Alloprevotella* | 0,75 | 0,23 | 0,0374 |
| Clostridia_unclassified | 0,18 | 0,74 | 0,0250 |
| Synergistaceae_unclassified | 0,11 | 0,66 | 0,0039 |
| Proteobacteria_unclassified | 0,53 | 0,10 | 0,0039 |
| *Mogibacterium* | 0,11 | 0,24 | 0,0039 |
| Clostridiaceae_1_unclassified | 0,20 | 0,00 | 0,0021 |
| *Bacteroides* | 0,00 | 0,18 | 0,0132 |
| *Succinivibrio* | 0,00 | 0,14 | 0,0021 |
| *Vampirovibrio* | 0,12 | 0,01 | 0,0037 |
| Coriobacteriia_unclassified | 0,09 | 0,02 | 0,0198 |
| *Clostridium*_sensu_stricto | 0,10 | 0,02 | 0,0237 |
| *Campylobacter* | 0,08 | 0,02 | 0,0240 |
| *Lysinibacillus* | 0,10 | 0,00 | 0,0222 |
| *Clostridium*_IV | 0,07 | 0,02 | 0,0242 |
| *Mailhella* | 0,06 | 0,02 | 0,0148 |
| Flexilinea_unclassified | 0,06 | 0,02 | 0,0159 |
| Eubacteriaceae_unclassified | 0,05 | 0,01 | 0,0079 |
| *Sporobacter* | 0,05 | 0,01 | 0,0190 |
| *Helicobacter* | 0,05 | 0,00 | 0,0108 |
| *Solibacillus* | 0,05 | 0,00 | 0,0069 |
| *Agathobacter* | 0,05 | 0,00 | 0,0222 |
| Planctomycetes_unclassified | 0,04 | 0,01 | 0,0048 |
| SR1_unclassified | 0,04 | 0,00 | 0,0250 |
| *Anaerosporobacter* | 0,04 | 0,00 | 0,0021 |
| *Anaeroplasma* | 0,03 | 0,00 | 0,0035 |
| *Lacrimispora* | 0,03 | 0,00 | 0,0074 |
| *Clostridium*_XlVa | 0,02 | 0,00 | 0,0021 |
| *Viridibacillus* | 0,02 | 0,00 | 0,0074 |
| *Anaerotignum* | 0,01 | 0,00 | 0,0019 |
| Desulfovibrionales_unclassified | 0,01 | 0,00 | 0,0071 |
| *Actinobacillus* | 0,01 | 0,00 | 0,0209 |
| *Pseudomonas* | 0,005 | 0,000 | 0,0190 |
